# Diversity and Risk Factors Associated with Multidrug and Methicillin-Resistant Staphylococci Isolated from Cats Admitted to a Veterinary Clinic in Eastern Province, Saudi Arabia

**DOI:** 10.3390/antibiotics10040367

**Published:** 2021-03-31

**Authors:** Ahmed Elmoslemany, Ibrahim Elsohaby, Mohammed Alorabi, Mohamed Alkafafy, Theeb Al-Marri, Ali Aldoweriej, Fanan A. Alaql, Abdullah Almubarak, Mahmoud Fayez

**Affiliations:** 1 Hygiene and Preventive Medicine Department, Faculty of Veterinary Medicine, Kafrelsheikh University, Kafr El-Sheikh 33516, Egypt; aelmoslemany@gmail.com; 2 Department of Animal Medicine, Faculty of Veterinary Medicine, Zagazig University, Zagazig City 44511, Egypt; 3 Department of Health Management, Atlantic Veterinary College, University of Prince Edward Island, Charlottetown, PE C1A 4P3, Canada; 4 Department of Biotechnology, College of Science, Taif University, P.O. Box 11099, Taif 21944, Saudi Arabia; maorabi@tu.edu.sa (M.A.); m.kafafy@tu.edu.sa (M.A.); 5 Al-Ahsa Veterinary Diagnostic Lab, Ministry of Environment, Water and Agriculture, Al-Ahsa 31982, Saudi Arabia; theep8@hotmail.com (T.A.-M.); Abd-20151@hotmail.com (A.A.); mahmoudfayez30@hotmail.com (M.F.); 6 Veterinary Health and Monitoring, Ministry of Environment, Water and Agriculture, Riyadh 11195, Saudi Arabia; Dr.alivet@mewa.gov.sa; 7 Department of Microbiology, College of Science, King Saud University, Riyadh 11451, Saudi Arabia; fanan.abdulaziz@gmail.com; 8 Department of Bacteriology, Veterinary Serum and Vaccine Research Institute, Ministry of Agriculture, Cairo 131, Egypt

**Keywords:** antimicrobial resistance, *Staphylococcus*, cats, methicillin-resistance, multidrug resistance, risk factors

## Abstract

Understanding the distribution, antimicrobial resistance (AMR), and risk factors associated with multidrug-resistant (MDR) and methicillin-resistant staphylococci (MRS) isolated from cats admitted to veterinary clinics may decrease the risk of MDR and MRS transmission to humans and other cats. As such, the objectives of this study were to investigate the diversity in *Staphylococcus* spp. recovered from different anatomical locations in healthy and diseased cats and to determine the occurrence of MDR and MRS spp. as well as possible risk factors associated with colonization in these cats. Five swabs were collected from the anus, skin, ear canal, conjunctival sac, and nares of each cat (209 healthy and 191 diseased) admitted to a veterinary clinic in Eastern Province, Saudi Arabia, between January and December 2018. Prior to sample collection, cat owners completed a questionnaire collecting information on cat demographics, health status, management, and antimicrobial usage. In total, 179 *Staphylococcus* isolates were recovered from healthy (*n* = 71) and diseased (*n* = 108) cats, including 94 (52.5%) coagulase-positive staphylococci (CoPS), and 85 (47.5%) coagulase-negative staphylococci (CoNS). Five *Staphylococcus* spp. were identified, namely, *Staphylococcus aureus*, *Staphylococcus pseudintermedius*, *Staphylococcus felis*, *Staphylococcus capitis*, and *Staphylococcus saprophyticus*. *Staphylococcus* isolates were most commonly resistant to penicillin (56.4%) and ciprofloxacin (25.7%); however, no isolate was resistant to clindamycin. Thirty (16.8%) *Staphylococcus* spp. (24 *S. aureus* and 6 *S. pseudintermedius*) isolates were MDR, with resistance to up to six different antibiotic classes. Only 17 (9.5%) *Staphylococcus* spp. (15 methicillin-resistant *S. aureus* and 2 methicillin-resistant *S. pseudintermedius*) harbored the *mecA* gene. Risk factor analysis showed that cats with a history of antibiotic therapy, those raised mainly indoors with a child, and those who visit a veterinary clinic for treatment were at higher risk of MDR and MRS colonization. In conclusion, MDR and MRS were common in healthy and diseased cats in Saudi Arabia. Thus, an effective antimicrobial stewardship program and further studies using a One Health approach are required to investigate the role of cats as vectors for AMR transmission to humans.

## 1. Introduction

Staphylococci are opportunistic bacteria that may colonize the skin, mucous membrane of the nasal cavity, conjunctival sac, throat, and anus [1,2]. *Staphylococci* are currently categorized on the basis of their ability to produce coagulase: coagulase-positive staphylococci (CoPS) or coagulase-negative staphylococci (CoNS). *Staphylococcus aureus* and *Staphylococcus pseudintermedius* are the most prevalent CoPS spp. isolated from humans and animals [3]. Numerous CoNS spp. have been isolated, such as *Staphylococcus saprophyticus*, *Staphylococcus capitis*, *Staphylococcus haemolyticus*, and *Staphylococcus felis* [4]. CoPS spp. have higher pathogenic potential than CoNS spp. [4,5]; this may be attributed to the association between CoNS spp. and both chronic and subacute infections [5]. Furthermore, CoNS spp. are less frequently involved in community-associated diseases but are one of the major nosocomial pathogens that impact human health [4,6].

In humans and animals, staphylococcal infections are generally treatable with topical or systemic antimicrobials [7]. However, the worldwide spread of antimicrobial resistance makes treatment with antimicrobial agents difficult [3]. In terms of antimicrobial resistance, methicillin-resistant staphylococci (MRS) are of particular importance because they are resistant to all the β-lactams antibiotics commonly used in animals and humans and thus have a major impact on public health [8,9].

Household pets, such as dogs and cats share a common environment with their owners and are treated with antimicrobial agents similar to those prescribed to humans. The close contact between pets and owners allows the transmission of zoonotic bacteria either directly through contact or indirectly through contamination of food and the environment [10]. Therefore, household pets are considered potential reservoirs of antimicrobial resistant zoonotic bacteria, such as methicillin-resistant *S. aureus* (MRSA), methicillin-resistant *S. pseudintermedius* (MRSP), and extended-spectrum β-lactamase- producing *Escherichia coli* [11,12,13]. Several studies have reported that the majority of MRSA found in pets originates from humans, either pet owners or veterinarians [3,14]; however, veterinarians are at higher risk of MRSA infection than pet owners [15]. Another study attributed pets as an occasional source of MRSA infection in pet owners [16]. Thus, it seems that MRSA can be transmitted between animals and humans in a bi-directional manner [17].

Information regarding antimicrobial use in household pets, risk factors, and transmission routes of antimicrobial resistant bacteria between humans and household pets is scarce [18]. Several studies have reported infections related to MRSA in dogs and cats [3,19,20]. Nonetheless, few studies have investigated the staphylococci populations in healthy cats, as well as infections due to MRSP and methicillin-resistant CoNS species in cats [17,21]. In Saudi Arabia, a large number of studies have focused on MRSA infection in farm animals, including cattle [22], goats [23], and camels [24]; however, to the author’s knowledge, no studies have investigated the diversity and multidrug resistance (MDR) of both CoPS and CoNS spp. in healthy and diseased cats. Therefore, this study aimed to (1) investigate the diversity in *Staphylococcus* spp. recovered from different anatomical locations in healthy and diseased cats; (2) determine the occurrence of MDR and MRS spp. recovered from healthy and diseased cats; and (3) determine the possible risk factors associated with colonization of MDR and MRS spp. recovered from healthy and diseased cats in Eastern Province, Saudi Arabia.

## 2. Results

### 2.1. Study Population

Between January and December 2018, a total of 2000 swabs were collected from 400 cats admitted to a veterinary clinic in Eastern Province, Saudi Arabia. The study population was 43.3% (173/400) male and 56.7% (227/400) female. The breeds of cats included in the study were Persian (*n* = 162), Siamese (*n* = 112), Himalayan (*n* = 59), Birman (*n* = 53), Egyptian Mau (*n* = 10), and Arabian Mau (*n* = 4). Most cats were admitted for vaccination and/or grooming (209/400), but other reasons for admission included clinical examination due to digestive distress (81/400), respiratory distress (39/400), eye discharge (59/400), ear discharge (61/400), and skin wound and/or abscess (64/400).

### 2.2. Staphylococcus spp. Distribution

**Figure 1:**
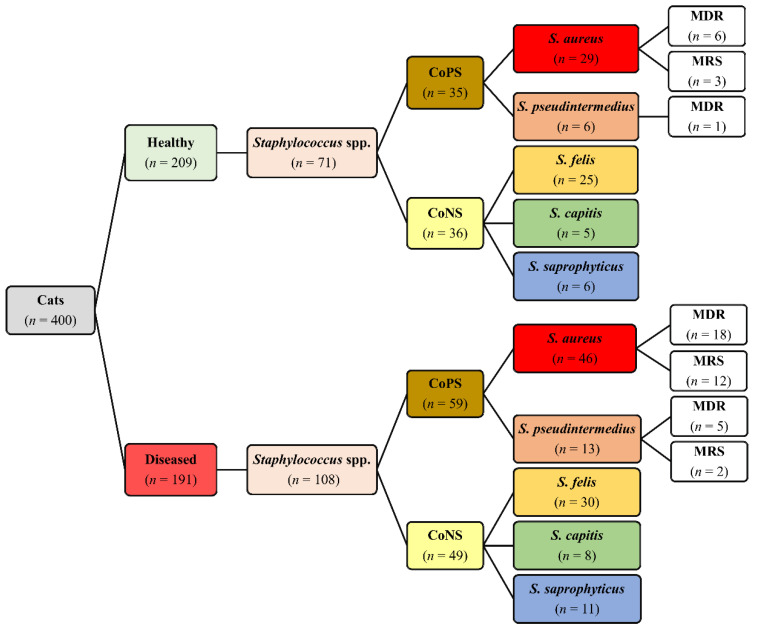
Distribution of *Staphylococcus* spp., multidrug resistance (MDR) and methicillin-resistant staphylococci (MRS) isolated from 400 healthy and diseased cats admitted to a veterinary clinic in Eastern Province, Saudi Arabia.

Out of the 400 cats, *Staphylococcus* isolates were recovered from 153 (38.3%) cats, including 61 (29.2%) healthy and 92 (48.2%) diseased cats. In total, 179 *Staphylococcus* isolates were recovered from healthy (71/179) and diseased (108/179) cats (Figure 1). Out of the 179 isolates, 94 (52.5%) were CoPS and 85 (47.5%) were CoNS. On the basis of 16S rRNA, five *Staphylococcus* spp. were identified, namely, *S. aureus*, *S. pseudintermedius*, *S. felis*, *S. capitis*, and *S. saprophyticus*, with GenBank accession numbers MK123464, MK123481, MK123404, MK127538 and MK127551, respectively. CoPS isolates included 75 (79.8%) *S. aureus* and 19 (20.2%) *S. pseudintermedius*, whereas CoNS isolates included 55 (64.7%) *S. felis*, 13 (15.3%) *S. capitis* and 17 (20.0%) *S. saprophyticus* (Figure 1). The distribution of *Staphylococcus* spp. based on anatomical locations in healthy and diseased cats is presented in Table 1. There are no significant differences in distribution of *Staphylococcus* spp. between diseased and healthy cats (*p* = 0.833), cat sex (*p* = 0.893) and breeds (*p* = 0.844).

### 2.3. Antimicrobial Susceptibility

The antimicrobial susceptibility test showed that 56.4% of *Staphylococcus* isolates were resistant to penicillin (PEN), and 25.7% were resistant to ciprofloxacin (CIP); however, no isolate was resistant to clindamycin (CLI). The rates of resistance to amikacin (AMK), tetracycline (TET), trimethoprim/sulfamethoxazole (SXT), gentamicin (GEN), and amoxicillin clavulanic acid (AMC) were 17.3%, 16.8%, 16.2%, 14.0% and 12.8%, respectively, and 9.5%, 8.9%, and 7.8% of *Staphylococcus* isolates showed resistance to cefoxitin (FOX), chloramphenicol (CHL), and erythromycin (ERY), respectively.

**Figure 2:**
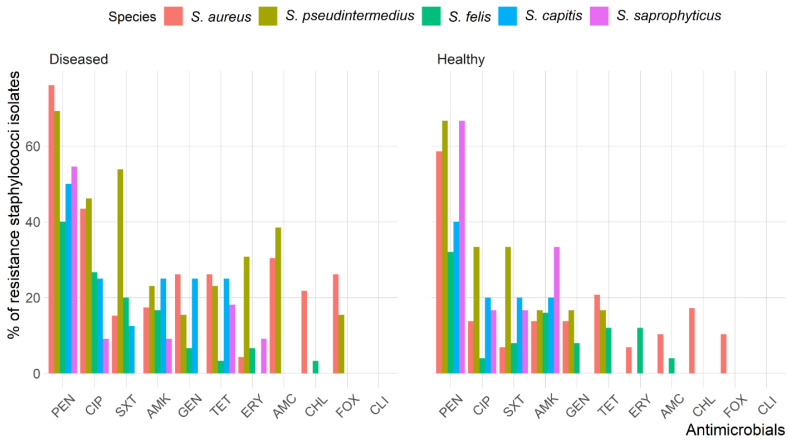
Frequency of antimicrobial resistance of *Staphylococcus* spp. recoverd from healthy and diseased cats.

**Figure 3:**
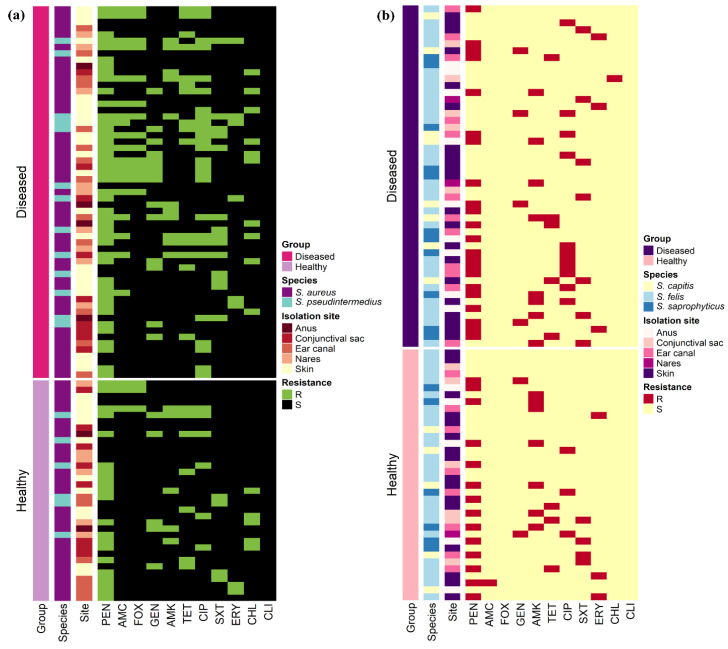
Heat map representation of the antimicrobial resistance patterns of (**a**) 94 coagulase-positive and (**b**) 85 coagulase-negative staphylococci isolates recovered from 400 healthy and diseased cats admitted to a veterinary clinic in Eastern Province, Saudi Arabia.

The antimicrobial resistance patterns of all *Staphylococcus* spp. isolated from healthy cats were slightly different than those isolated from diseased cats except for *S. aureus* (Figure 2). The frequency of isolates resistant to AMC (odds ratio (OR): 3.6, 95% confidence interval (CI): 1.2–11.0) and CIP (OR: 3.6, 95% CI: 1.6–8.1) were significantly greater in diseased cats than in healthy cats; however, there were no differences (*p* > 0.05) between diseased and healthy cats for the other antimicrobials. Figure 3 shows the antimicrobial resistance patterns of CoPS and CoNS isolates recovered from the different anatomical locations in healthy and diseased cats.

**Figure 4:**
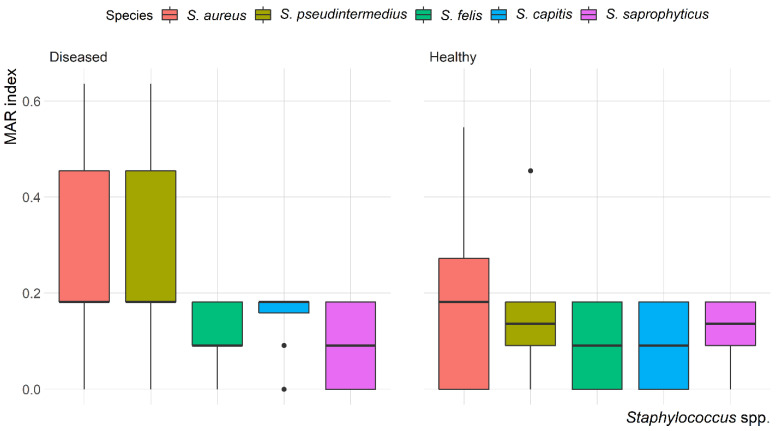
Box and whisker plot of multiple antibiotic resistance (MAR) index among *Staphylococcus* spp. recovered from different anatomical locations in healthy and diseased cats.

The mean multiple antibiotic resistance (MAR) index for *Staphylococcus* isolates was 0.22, ranging from 0.09 to 0.64. The highest MAR index (0.64) was found in *S. aureus* and *S. pseudintermedius* isolated from the skin of diseased cats. Most of the isolates (58.7%) showed a MAR index < 0.2. Variation in the MAR index of *Staphylococcus* spp. recovered from healthy and diseased cats is demonstrated in Figure 4. There was a significant (*p* = 0.003) difference in the MAR index of *S. aureus* recovered from healthy and diseased cats; however, there was no difference in the MAR index of *S. pseudintermedius*, *S. felis*, *S. capitis* and *S. saprophyticus* recovered from healthy and diseased cats. 

**Figure 5:**
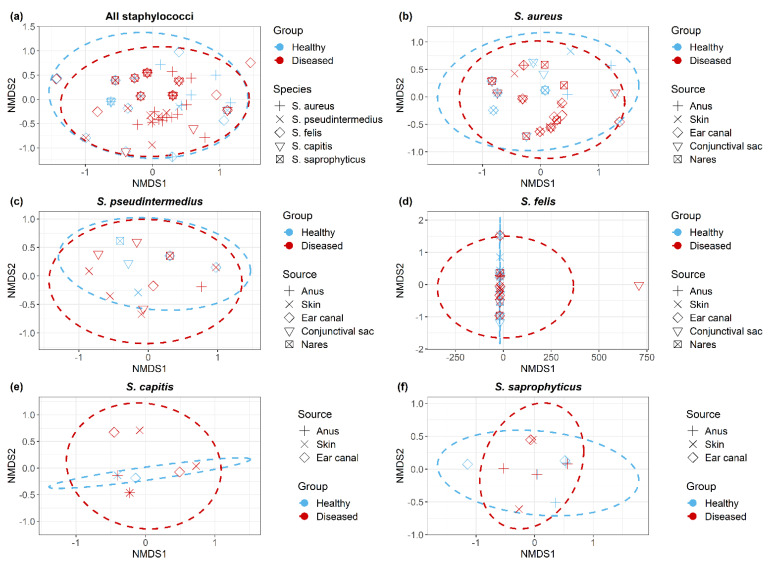
Non-metric multidimensional scaling ordination (NMDS) of antimicrobial resistant *Staphylococcus* isolates (**a**) across all *Staphylococcus* spp., (**b**) *S. aureus*, (**c**) *S. pseudintermedius*, (**d**) *S. felis*, (**e**) *S. capitis* and (**f**) *S. saprophyticus*. Dotted lines indicate clustering of isolates corresponds to cat group.

Non-metric multidimensional scaling (NMDS) plots demonstrated all *Staphylococcus* isolates (Figure 5a); within each *Staphylococcus* spp. (Figure 5b–f), isolates clustered according to the cat group (healthy/diseased) based on their antimicrobial resistance profiles (resistant/sensitive). *Staphylococcus* spp. isolated from healthy and diseased cats had similar antimicrobial profiles, but there were no significant differences between *S. aureus* isolates recovered from different anatomical locations in diseased cats.

### 2.4. MDR and MRS Isolates

Thirty (16.8%) *Staphylococcus* spp. (24 *S. aureus* and 6 *S. pseudintermedius*) were MDR isolates (Table 2), with resistance to up to six different antibiotic classes. The minimum inhibitory concentration of a representative number of MDR *Staphylococcus* spp. isolates are presented in Supplementary Table S1. In total, 17 (9.5%) *Staphylococcus* spp. (15 MRSA and 2 MRSP) were resistant to FOX, OXA and harbored the *mecA* gene (Table 2). Fourteen (82.4%) MRS isolates were recovered from diseased cats, and only three (17.6%) MRS isolates were recovered from healthy cats (Table 3). No MRSP isolates were found in healthy cats. MRSA ST80 and ST5 were the most prevalent isolates in healthy and diseased cats, respectively. On the other hand, MRSP ST71 was the only MRSP sequence type detected in any cats. The *PVL* genes (*lukS-PV* and *lukF-PV*) were detected in four (26.7%) MRSA isolates.

### 2.5. Risk Factors

Table 4 shows the univariable association (*p* < 0.25) between independent variables and each of the dependent variables (MDR and MRS). Both MDR and MRS were positively associated with a number of family characteristics, namely, the use of antibiotic therapy, the presence of acne, hospitalization of a family member, and having a child at home, in addition to previous and recent antibiotic therapy for the cat and visiting a veterinary clinic for treatment rather than vaccination. On the other hand, cats living partially indoors and outdoors had a lower risk of MDR and MRS compared with indoors living cats.

Table 5 shows the final multivariable logistic regression model for factors associated with MDR and MRS. The odds of MDR were approximately 9 and 6 times higher when the antimicrobial was previously used as treatment for the family and cat, respectively. The cat’s family having a child at home was also associated with higher odds of MDR (OR = 4.3). The odds of MDR were 3.6 times higher if the purpose of the veterinary clinic visit were to receive treatment rather than vaccination or grooming. Finally, indoor–outdoor cats had lower odds of MDR (OR = 0.3) compared with indoor-only cats. The model for MRS produced a similar set of predictors as the MDR model; this model also showed a higher risk (OR = 15.9) of MRS when a family member had acne.

## 3. Discussion

### 3.1. Staphylococcus spp. Distribution

This study demonstrated that many healthy cats (29.2%) are carriers of *Staphylococcus* spp.; this proportion was higher in diseased cats (48.2%). Similar studies have reported that *Staphylococcus* spp. are more frequently isolated in diseased cats than in healthy cats [25,26]. In this study, *Staphylococcus* spp. was recovered from various anatomical locations, including the skin, anus, conjunctival sac, ear canal, and nares, which is consistent with previous reports [27,28]. This distribution is common as *Staphylococcus* spp. are among the normal bacterial flora of various body sites.

In this study, CoPS spp. were isolated more often than CoNS spp.; however, other studies have reported CoNS spp. to be the most prevalent *Staphylococcus* in cats and dogs admitted to veterinary clinics in Canada and South Korea [28,29,30]. In this study, *S. aureus* was the most frequently identified *Staphylococcus* spp. in both healthy and diseased cats, followed by *S. felis*; this is in disagreement with previously published studies [26,31,32], which reported CoNS as the most frequently occurring species in healthy and diseased cats. *S. aureus* is part of the normal bacterial flora in cats, especially those who are kept in close contact with their owners, which may explain the high occurrence of *S. aureus* in this study [33]. Furthermore, *S. aureus* is thought to be involved in <5% of skin infections [29,34,35], but in this work, 38.7% (29/75) of *S. aureus* isolates were recovered from skin swabs. Other studies have reported *S. felis* [30] and *S. pseudintermedius* [31,36] as the most common *Staphylococcus* spp. in cats, regardless of their health.

### 3.2. Antimicrobial Susceptibility

The antimicrobial susceptibility test demonstrated the effectiveness of different antimicrobial agents, including CLI, FOX, CHL, and ERY, and identified the best initial choices for the treatment of *Staphylococcus* infection in diseased and healthy cats; the results of this susceptibility test confirmed the results previous studies [31,32]. In the present work, a high proportion of *Staphylococcus* isolates were resistant to PEN (56.4%) and CIP (25.7%). Results from other countries show wide variation in *Staphylococcus* resistance patterns, but PEN and CIP are generally less effective for treating *Staphylococcus* infections in cats [19,31,32]. The *Staphylococcus* isolates recovered from diseased cats were resistant to more antimicrobial agents than those recovered from healthy cats, with significant differences in resistance to AMC and CIP. Resistance to these agents, which are used extensively in veterinary clinics, has been reported to be common among staphylococci from diseased cats [37,38].

In this study, MDR *Staphylococcus* isolates were recovered from healthy and diseased cats. Thirty (16.8%) of the isolates were resistant to more than three antimicrobial classes; however, only 17 of the MDR isolates were associated with the presence of the *mecA* gene (MRS). This result is similar to that of Gandolfi-Decristophoris et al. [19], who reported that MDR was not always associated with the presence of the *mecA* gene. Interestingly, resistance to PEN, AMC, FOX, and CIP was associated with MRS isolates; this association is relevant and may reflect the higher exposure of MRS isolates to these agents. The high rates of MRS isolate resistance to PEN, AMC, FOX, and CIP is alarming as they are the key antimicrobials used for the decolonization of MRSA [4].

The MRSA isolates recovered in this study were linked to one clonal complex (CC8; ST 8 and 239) and three singleton clones (ST80, 22 and 5). MRSA-ST80 has emerged as an important pathogen in community and hospital infections in many Middle Eastern countries. In Saudi Arabia, two studies reported the same proportions of MRSA-ST80 in nasal carriage and infections in hospitals [39,40]. The ability of MRSA-ST80 to disseminate in the community has been reported [41,42]. Furthermore, the transmission of MRSA-ST80 among different host species in the context of households and veterinary practices has been described by Drougka et al. [41], who reported that dogs and cats can be reservoir of MRSA-ST80, causing severe infection in humans. In Saudi Arabia, the pandemic Vienna/Hungarian/Brazilian clone (CC8/ST239-III) and its variants continue to circulate in Dammam and Riyadh [43]. Furthermore, the hospital-associated MRSA-ST239-III was the prevalent clone detected in most of the studies performed in Saudi Arabia between 2001 and 2013 [44]. The pediatric clone (CC5) has also identified in rare cases in Saudi Arabian hospitals [44]. Results obtained from this study showed that only four MRSA isolates were *PVL* gene positive. The *PVL* gene is considered as a stable genetic marker for community-associated MRSA [45] and has been associated with necrotic skin lesions and community-acquired necrotic pneumonia [46].

### 3.3. Risk Factors

The identification of risk factors for MDR and MRS staphylococci colonization in pets is essential to controlling infection and transmission between humans and animals. Cats carrying MRSA represent a potential risk to human health because close contact between cats and owners can facilitate pathogen transfer. In this study, cats visiting the veterinary clinic for treatment had a higher risk of MDR and MRS compared with those visiting the veterinary clinic for vaccination and/or grooming. Similar results have been reported for MRSA [15] and MRS [32] in dogs and cats. Additionally, previous antibiotics treatment, for either the cat or family members, was associated with a higher risk of MDR and MRS. Other studies have also identified previous antimicrobial therapy as a risk factor for the development of colonization by MRS [33,47].

Antibiotic therapy may support MRS colonization by suppressing other competing bacterial infections. There was a positive association between MDR or MRS and the presence of a child at home. Compared with adult, children are more likely to handle animals extensively and touch their faces or mouths, which increase the risk of disease transmission. A recent study on community-associated MRSA in children showed that pets whose primary caretaker was MRSA colonized were more likely to be MRSA colonized than pets whose primary caretaker was not MRSA-colonized (50% vs. 4%) [48]. Cats living indoors showed a higher risk of MDR or MRS than those living indoors–outdoors. This association between indoor living and increased risk of MDR or MRS may be due to the increased close contact between humans and cats in indoor settings or related to indoor cats being more commonly located in urban areas, with greater access to veterinary care and, consequently, more antibiotic prescriptions. The reason for the veterinary clinic visit was a risk factor for MDR and MRS colonization, which is in accordance with the results obtained from previous studies [32,47,49]. Like previous studies, we did not find a significant association between MDR and MRS colonization and the cat’s sex, breed, or type of diet [50].

### 3.4. Limitations

It should be noted that our study has limitations. The study design, including sampling and risk factor assessment at a single point in time, cannot be used to establish a temporal relationship between MDR or MRS and the different risk factors; however, our results provide baseline information for control measures as well as for the design of future studies. Cat owners reported all the risk factors in the study, so recall bias may affect the information that was provided; it likely had a minimal impact, and we do not consider this bias to be significant.

## 4. Materials and Methods

### 4.1. Study Population and Animals

The study was conducted between January and December 2018 and included 400 cats admitted to a veterinary clinic in Eastern Province, Saudi Arabia. Cats included in the study were divided into two groups based on the reason for their visit to the clinic: (a) healthy cats (*n* = 209) included apparently healthy cats that were admitted to the clinic for vaccination and/or grooming; (b) diseased cats (*n* = 191) included cats that were admitted for clinical examination and had one or more of the following clinical signs: conjunctivitis, otitis, diarrhea, skin wound, abscess, and/or respiratory signs. Cat recruitment was on a voluntary basis. Cat owners were asked to provide a written informed consent prior to their cats being sampled.

### 4.2. Sample and Data Collection

Five swabs were collected from the anus, skin, ear canal, conjunctival sac, and nares of each cat. In total, 2000 swabs were collected by professional veterinarians conducted in accordance with the Local Ethics Committee for animal research in Saudi Arabia. Each swab was kept in a sterile tube containing 2 mL of liquid brain–heart infusion broth (BHI: Difco) and then transported cooled at 4 °C to the laboratory for later analysis. Prior to sample collection, owners completed a questionnaire collecting information about demographics, health status, management, and antimicrobial usage; this information is presented in Supplementary Table S2.

### 4.3. Staphylococcus spp. Isolation

Tubes containing swabs and 2 mL of BHI were incubated aerobically at 37 °C for 18 h. A 10 µL aliquot of the broth was streaked onto Baird–Parker agar supplemented with egg yolk tellurite, mannitol salt agar, and blood agar (Oxoid, Basingstoke, Hampshire, UK) and then incubated aerobically at 37 °C for 24 h. Morphologically typical staphylococcal colonies were purified on 5% sheep blood agar and verified by Gram staining, plus a coagulase and catalase test. Biochemically identified *Staphylococcus* isolates were defined to the species level with the VITEK^®^ 2 COMPACT system (bioMerieux, Marcy l’Etoile, France) using Gram-positive cards, following manufacturer guidelines. Presumptively identified staphylococcal colonies were stored in 40% glycerol saline at −70 °C for further investigation.

### 4.4. Staphylococcus spp. Identification

According to manufacturer guidelines, genetic DNA from *Staphylococcus* isolates was extracted from an overnight culture using the QIAamp DNA mini kit (Qiagen SA, Courtaboeuf, France). Extracted DNA was subjected to PCR using primers specific for *Staphylococcus* 16S rRNA and reaction conditions described by Jaffe et al. [51]. The PCR products were verified on 1.5% agarose gel and then sequenced using Genetic Analyzer 3500 (Applied Biosystems, Foster City, CA, USA). The 16S rRNA sequences were compared with sequences deposited in the GenBank database using the BLAST algorithm. Identification of *Staphylococcus* spp. was deemed reliable if sequence similarities with reference genes were >98%.

### 4.5. Antimicrobial Susceptibility Testing

The antimicrobial susceptibility of *Staphylococcus* isolates was determined using the disk diffusion method. Eleven different antimicrobial agents that are widely used for companion animals in Saudi Arabia were tested: penicillin (PEN: 10 units), amoxicillin clavulanic acid (AMC: 20/10 μg), cefoxitin (FOX: 30 µg), gentamicin (GEN: 10 μg), amikacin (AMK: 30 μg), tetracycline (TET: 30 µg), ciprofloxacin (CIP: 5 μg), trimethoprim/sulfamethoxazole (SXT: 1.25/23.75 μg), erythromycin (ERY: 15 μg), chloramphenicol (CHL: 30 μg), and clindamycin (CLI: 2 μg). These antimicrobial agents were purchased from Oxoid (Basingstoke, Hampshire, UK). Results were interpreted according to the Clinical and Laboratory Standards Institute (CLSI) [52] and European Committee on Antimicrobial Susceptibility Testing (EUCAST) guidelines for antimicrobial susceptibility tests [53]. *Staphylococcus aureus* reference strain ATCC 29737 was included in each run to assess the reliability of the method. MDR was defined as resistance to at least three antimicrobial agents belonging to three different antibiotic classes [54]. The multiple antibiotic resistance (MAR) index was calculated as the ratio of the number of antibiotics to which the isolate displayed resistance to the number of antibiotics to which the isolate had been evaluated for susceptibility [55].

### 4.6. MRS spp. Identification

The standard disk diffusion method was used to screen all *Staphylococcus* isolates for phenotypic methicillin resistance. The antimicrobial agent FOX (30 µg) and oxacillin (OXA: 1 µg) was used as described by CLSI [52]. *Staphylococcus* isolates that showed phenotypic resistance to FOX and OXA were investigated for the presence of the *mecA* gene, encoding methicillin resistance, using PCR, as described previously by Murakami et al. [56]. Multilocus sequence typing (MLST) of *mecA*-positive isolates was performed as previously described [57,58]. The sequence type (ST) and allelic profile of methicillin-resistant isolates (MRSA and MRSP) were assigned by comparing the database on the MLST website (https://pubmlst.org/, accessed on 30 March 2021). MRSA isolates were also screened for the presence of the virulence gene Panton–Valentine leucocidin (*PVL*). Two different alleles of the *PVL* genes *lukS-PV* and *lukF-PV*, were investigated according to the method described by Otokunefor et al. [59].

### 4.7. Data Analysis

Data were visualized with R software (R Core Team, 2019; version 3.5.3). The R package “Complex-Heatmap” was used to build a heatmap based on the antimicrobial resistance profiles of each isolate [60]. Associations between *Staphylococcus* spp. isolates and cat condition (healthy vs. diseased), sex, and breeds were assessed using the Chi-square and Fisher’s Exact tests. Non-metric multidimensional scaling (NMDS) [61] was performed using the metaMDS function of the vegan package to compare the dissimilarity of antimicrobial resistance profiles, using the Bray–Curtis distance between isolates across all *Staphylococcus* spp. and within each species. Permutational multivariate analysis of variance (PERMANOVA) [62] was employed using the adonis function of the vegan package to test whether the *Staphylococcus* isolates recovered from different anatomical locations in healthy and diseased cats had equivalent antimicrobial resistance profiles. *p*-values for PERMANOVA test statistics (F) were obtained using 999 permutations.

The association between the dependent variables (1—MDR (1 = yes vs. 0 = no), 2—MRS, (1 = yes vs. 0 = no)) and each of the independent variables (Table S1) was assessed using univariable logistic regression analyses. All variables with a *p* < 0.25 in the initial univariable analysis were checked for multicollinearity using Spearman’s rank-order correlation statistics. The final multivariable logistic model was built using backward stepwise elimination at *p* < 0.05. The fit of the final model was assessed using the Hosmer–Lemeshow test and the predictive ability was assessed using the receiver operating characteristic curve [63].

## 5. Conclusions

This is the first comprehensive study to investigate antimicrobial resistance and risk factors associated with *Staphylococcus* spp. colonization in healthy and diseased cats admitted to a veterinary clinic in Saudi Arabia. *Staphylococcus* spp. were isolated from 38.3% of the cats, and CoPS (*S. aureus*) were the most commonly isolated species in both healthy and diseased cats. The threat of MDR staphylococci is steadily increasing in Saudi Arabia. Our results show that MDR and MRS were common in both healthy and diseased cats, highlighting the importance of monitoring antimicrobial use and resistance in pets. The isolation of MRSA-ST80 from cats belonging to community-associated clonal lineages emphasizes the cross-transmission of MRSA between cats and humans. An effective antimicrobial stewardship program and further studies using a One Health approach may be required to investigate the role of cats as vectors for transmission of antimicrobial resistance to humans.

## Figures and Tables

**Table 1:** Number of *Staphylococcus* spp. isolated from 400 healthy and diseased cats admitted to a veterinary clinic in Eastern Province, Saudi Arabia.

Anatomical Locations	No. of Isolates(%)	No. (%) of Coagulase-Positive Staphylococci		No. (%) of Coagulase-Negative Staphylococci		
		*S. aureus*	*S. pseudintermedius*	*S. felis*	*S. capitis*	*S. saprophyticus*
		**I—Healthy Cats (*n* = 209)**				
Anus	7 (3.4)	2 (28.6)	0 (0.0)	1 (14.3)	1 (14.3)	3 (42.9)
Skin	28 (13.4)	10 (35.7)	2 (7.1)	13 (46.4)	2 (7.1)	1 (3.6)
Ear canal	15 (7.2)	5 (33.3)	2 (13.3)	4 (26.7)	2 (13.3)	2 (13.3)
Conjunctival sac	14 (6.7)	7 (50.0)	1 (7.1)	6 (42.9)	0 (0.0)	0 (0.0)
Nares	7 (3.4)	5 (71.4)	1 (14.3)	1 (14.3)	0 (0.0)	0 (0.0)
Total	71 (34.0)	29 (40.8)	6 (8.5)	25 (35.2)	5 (7.0)	6 (8.5)
		**II—Diseased Cats (*n* = 191)**				
Anus	13 (6.8)	3 (23.1)	1 (7.7)	3 (23.1)	2 (15.4)	4 (30.8)
Skin	45 (23.6)	19 (42.2)	5 (11.1)	13 (28.9)	4 (8.9)	4 (8.9)
Ear canal	25 (13.1)	11 (44.0)	2 (8.0)	8 (32.0)	2 (8.0)	2 (8.0)
Conjunctival sac	14 (7.3)	6 (42.9)	3 (21.4)	4 (28.6)	0 (0.0)	1 (7.1)
Nares	11 (5.8)	7 (63.6)	2 (18.2)	2 (18.2)	0 (0.0)	0 (0.0)
Total	108 (56.5)	46 (42.6)	13 (12.0)	30 (27.8)	8 (7.4)	11 (10.2)

**Table 2:** Number of multidrug resistance (MDR) and methicillin-resistant staphylococci (MRS) isolates recovered from healthy and diseased cats admitted to a veterinary clinic in Eastern Province, Saudi Arabia.

*Staphylococcus* spp.	*n*	No. of MDR		Total	No. of MRS		Total
		Healthy	Diseased		Healthy	Diseased	
Coagulase positive	94	7/35	23/59	30/94	3/35	14/59	17/94
*S. aureus*	75	6/29	18/46	24/75	3/29	12/46	15/75
*S. pseudintermedius*	19	1/6	5/13	6/19	0/6	2/13	2/19
Coagulase negative	85	0/36	0/49	0/85	0/36	0/49	0/85
*S. felis*	55	0/25	0/30	0/55	0/25	0/30	0/55
*S. capitis*	13	0/5	0/8	0/13	0/5	0/8	0/13
*S. saprophyticus*	17	0/6	0/11	0/17	0/6	0/11	0/17
Total	179	7/71	23/108	30/179	3/71	14/108	17/179

**Table 3:** Multilocus sequence typing (MLST) and antimicrobial resistance patterns of methicillin-resistant *S. aureus* (MRSA) and *S. pseudintermedius* (MRSP) isolates recovered from healthy and diseased cats admitted to a veterinary clinic in Eastern Province, Saudi Arabia.

Cat ID	Group	Anatomical Locations	Species	Sequence Type	Genotype ^1^		Antimicrobial Resistance Patterns	MAR ^2^
					*lukS*	*lukF*		
3	Healthy	Nares	MRSA	80	+	+	PEN, AMC, FOX	0.27
5	Healthy	Conjunctival sac	MRSA	80	+	+	PEN, AMC, FOX	0.27
15	Diseased	Skin	MRSA	22	–	–	PEN, AMC, FOX, TET, CIP	0.45
16	Diseased	Skin	MRSA	22	–	–	PEN, AMC, FOX, TET, CIP	0.45
34	Healthy	Skin	MRSA	239	–	–	PEN, AMC, FOX, AMK, TET, CIP	0.55
40	Diseased	Nares	MRSA	239	–	–	PEN, AMC, FOX, AMK, TET, CIP	0.55
57	Diseased	Ear canal	MRSA	239	–	–	PEN, AMC, FOX, AMK, TET, CIP	0.55
70	Diseased	Skin	MRSA	80	+	+	PEN, AMC, FOX	0.27
92	Diseased	Skin	MRSA	8	–	–	PEN, AMC, FOX, CIP, SXT	0.45
102	Diseased	Ear canal	MRSA	8	–	–	PEN, AMC, FOX, CIP, SXT	0.45
109	Diseased	Ear canal	MRSA	5	–	–	PEN, AMC, FOX, GEN, CIP, CHL	0.55
118	Diseased	Conjunctival sac	MRSA	5	–	–	PEN, AMC, FOX, GEN, CIP	0.45
125	Diseased	Skin	MRSA	5	–	–	PEN, AMC, FOX, GEN, CIP	0.45
133	Diseased	Ear canal	MRSA	5	–	–	PEN, AMC, FOX, GEN, CIP	0.45
139	Diseased	Nares	MRSA	80	+	+	PEN, AMC, FOX	0.27
27	Diseased	Skin	MRSP	71	--	--	PEN, AMC, OXA, AMK, CIP, SXT, ERY	0.64
82	Diseased	Skin	MRSP	71	--	--	PEN, AMC, OXA, AMK, CIP, SXT, ERY	0.64

^1^ + = PVL genes positive; – = PVL genes negative; -- = not tested. ^2^ MAR = multiple antibiotic resistance index.

**Table 4:** Univariable analysis of risk factors associated with multidrug resistance (MDR) and methicillin-resistant staphylococci (MRS) isolates recovered from 400 healthy and diseased cats admitted to a veterinary clinic in Eastern Province, Saudi Arabia.

Factors	MDR		MRS	
	Odds Ratio	*p*-Value	Odds Ratio	*p*-Value
Family use antimicrobials				
No	1.00 (ref.)		1.00 (ref.)	
Yes	6.0	0.000	18.2	0.000
Family member with acne				
No	1.00 (ref.)		1.00 (ref.)	
Yes	3.0	0.004	20.4	0.000
Hospitalization				
No	1.00 (ref.)		1.00 (ref.)	
Yes	4.6	0.000	8.3	0.000
Previous antimicrobial use for cat				
No	1.00 (ref.)		1.00 (ref.)	
Yes	5.5	0.000	7.4	0.002
Type of previously used antimicrobials				
Non	1.00 (ref.)	0.000	1.00 (ref.)	0.000
Cefalexin	4.4	0.006	3.6	0.121
Ampicillin	10.2	0.001	24.1	0.000
Amoxicillin	2.8	0.223	6.5	0.045
Current antimicrobials use for cat				
No	1.00 (ref.)		1.00 (ref.)	
Yes	12.2	0.000	29.1	0.000
Child at home				
No	1.00 (ref.)		1.00 (ref.)	
Yes	3.8	0.003	3.6	0.017
Cat living				
Indoors	1.00 (ref.)	0.008	1.00 (ref.)	0.005
Indoors-outdoors	0.3	0.002	0.2	0.002
Family living				
Urban	1.00 (ref.)	0.012	1.00 (ref.)	0.091
Countryside	0.2	0.043	0.2	0.138
Apartment	0.3	0.016	0.4	0.142
Reason being at clinic				
Vaccination and/or grooming	1.00 (ref.)		1.00 (ref.)	
Treatment	4.0	0.002	5.4	0.009
Cat care				
Adult male	1.00 (ref.)	0.012	1.00 (ref.)	0.003
Adult female	0.3	0.002	0.1	0.003
Child	0.4	0.313	0.5	0.530
All family	0.3	0.057	0.1	0.043

**Table 5:** Multivariable logistic regression analysis of risk factors associated with multidrug resistance (MDR) and methicillin-resistant staphylococci (MRS) isolates recovered from 400 healthy and diseased cats admitted to a veterinary clinic in Eastern Province, Saudi Arabia.

Factors	MDR		MRS	
	OR (95% CI) ^1^	*p*-Value	OR (95% CI) ^1^	*p*-Value
Family use antimicrobials				
No	1.00 (ref.)		1.00 (ref.)	
Yes	8.8 (3.47–22.30)	0.000	11.9 (2.48–57.46)	0.002
Family member with acne				
No	-	-	1.00 (ref.)	
Yes	-	-	15.9 (2.64–95.45)	0.003
Previous antimicrobial use for cat				
No	1.00 (ref.)		1.00 (ref.)	
Yes	6.1 (2.21–16.60)	0.000	12.4 (2.56–59.67)	0.034
Child at home				
No	1.00 (ref.)		1.00 (ref.)	
Yes	4.3 (1.63–11.54)	0.003	6.9 (1.46–32.43)	0.015
Cat living				
Indoors	1.00 (ref.)		1.00 (ref.)	
Indoors–outdoors	0.29 (0.12–0.69)	0.006	0.15 (0.04-–0.65)	0.011
Reason being at clinic				
Vaccination and/or grooming	1.00 (ref.)		1.00 (ref.)	
Treatment	3.6 (1.34–9.61)	0.011	5.4 (1.14–25.88)	0.034
**_cons**	0.001 (0.000–0.010)	0.000	0.00 (0.000–0.000)	0.000

^1^ OR: odds ratio; CI: confidence interval.

## Data Availability

The data presented in this study are available on request from the corresponding author.

## Supplementary Materials

The following are available online at https://www.mdpi.com/article/10.3390/antibiotics10040367/s1, Table S1: Description and categories of the collected variables. Table S2. Description and categories of the collected variables.
