# Supplementary material for: Diversity and Risk Factors Associated with Multidrug and Methicillin-Resistant Staphylococci Isolated from Cats Admitted to a Veterinary Clinic in Eastern Province, Saudi Arabia
Source: Antibiotics (Basel). 2021 Mar 31;10(4):367. doi: 10.3390/antibiotics10040367 (PMC8066541; doi:10.3390/antibiotics10040367)
Supplement: Supplementary file 1 [file antibiotics-10-00367-s001.pdf]

Table S1. Minimum inhibitory concentration (MIC) of a representative number of multidrug resistant *Staphylococcus* spp. isolates.

Cat ID	*Staphylococcus* spp.	Group	Source	PEN	AMC	OXA/ FOX	GEN	AMK	TET	CIP	SXT	ERY	CHL	CLI
85	*S. pseudintermedius*	Diseased	Skin	4	1	0.125	32	64	32	8	2	0.25	2	0.25
27	*S. pseudintermedius*	Diseased	Skin	2	16	4	2	128	2	16	32	16	1	0.25
82	*S. pseudintermedius*	Diseased	Skin	8	16	2	1	128	1	16	64	16	2	0.125
89	*S. pseudintermedius*	Diseased	Ear	16	2	0.125	64	8	64	8	32	0.125	4	0.125
34	*S. aureus*	Healthy	Skin	16	32	32	2	32	32	1	2	0.25	4	0.25
109	*S. aureus*	Diseased	Ear	16	32	8	32	8	2	1	2	0.25	32	0.25
183	*S. aureus*	Diseased	Skin	2	32	2	2	64	1	1	16	0.25	64	0.25
102	*S. aureus*	Diseased	Ear	32	32	32	2	8	2	1	1	0.125	7	0.125

PEN = penicillin; AMC = amoxicillin clavulanic acid, FOX = cefoxitin; GEN = gentamicin; AMK = amikacin; TET = tetracycline; CIP = ciprofloxacin; SXT = trimethoprim/sulfamethoxazole; ERY = erythromycin; CHL = chloramphenicol; CLI = clindamycin.

Table S2. Description and categories of the collected variables.

Factors	Categories	Frequency	%
**Cat sex**			
	Female	227	56.75
	Male	173	43.25
**Cat breed**			
	Himalayan	59	14.75
	Persian	162	40.50
	Birman	53	13.25
	Siamese	112	28.00
	Egyptian Mau	10	2.50
	Arabian Mau	4	1.00
**Family use antimicrobials**			
	No	308	77.0
	Yes	92	23.0
**Family member with acne**			
	No	282	70.5
	Yes	118	29.5
**Hospitalization**			
	No	334	83.5
	Yes	66	16.5
**Previous antimicrobial use for cat**			
	No	238	59.5
	Yes	162	40.5
**Type of previously used antimicrobials**			
	Non	236	59.50
	Cefalexin	68	17.00
	Ampicillin	17	4.25
	Amoxicillin	26	6.50
	Metronidazole	9	2.25
	Enrofloxacin	10	2.50
	Cefotaxime	9	2.25
	Clindamycin	23	5.75
**Current antimicrobials use for cat**			
	No	384	96.0
	Yes	16	4.0
**Type of current used antimicrobials**			
	Non	384	96.00
	Cefalexin	5	1.25
	Ampicillin	3	0.75
	Amoxicillin	4	1.00
	Enrofloxacin	2	0.50
	Cefotaxime	1	0.25
	Clindamycin	1	0.25
**Child at home**			
	No	205	51.25
	Yes	195	48.75
**Cat living**			
	Indoors	133	33.25

	Indoors-outdoors	267	66.75
**Family living**			
	Urban	208	52.0
	Countryside	72	18.0
	Apartment	120	30.0
**Reason being at clinic**			
	Vaccination and/or grooming	209	52.25
	Treatment	191	47.75
**Cat care**			
	Adult male	116	29.0
	Adult female	192	48.0
	Child	18	4.5
	All family	74	18.5
**diet type**			
	Dry	263	65.75
	Semi-moist	2	0.50
	Wet	53	13.25
	Raw uncooked	35	8.75
	Home available	47	11.75
**Total**		**400**	
